# White matter integrity is associated with alcohol cue reactivity in heavy drinkers

**DOI:** 10.1002/brb3.204

**Published:** 2013-12-29

**Authors:** Mollie A Monnig, Rachel E Thayer, Arvind Caprihan, Eric D Claus, Ronald A Yeo, Vince D Calhoun, Kent E Hutchison

**Affiliations:** 1 Mind Research NetworkAlbuquerque, 87106, New Mexico; 2 University of New MexicoAlbuquerque, 87106, New Mexico; 3 University of ColoradoBoulder, 80309, Colorado

**Keywords:** Alcohol use disorders, diffusion tensor imaging, functional magnetic resonance imaging, tract-based spatial statistics, white matter

## Abstract

Neuroimaging studies have shown that white matter damage accompanies excessive alcohol use, but the functional correlates of alcohol-related white matter disruption remain unknown. This study applied tract-based spatial statistics (TBSS) to diffusion tensor imaging (DTI) data from 332 heavy drinkers (mean age = 31.2 ± 9.4; 31% female) to obtain averaged fractional anisotropy (FA) values of 18 white matter tracts. Statistical analyses examined correlations of FA values with blood-oxygenation-level-dependent (BOLD) response to an alcohol taste cue, measured with functional magnetic resonance imaging (fMRI). FA values of nine white matter tracts (anterior corona radiata, body of corpus callosum, cingulate gyrus, external capsule, fornix, inferior frontooccipital fasciculus, posterior corona radiata, retrolenticular limb of internal capsule, and superior longitudinal fasciculus) were significantly, negatively correlated with BOLD activation in medial frontal gyrus, parahippocampal gyrus, fusiform gyrus, cingulum, thalamus, caudate, putamen, insula, and cerebellum. The inverse relation between white matter integrity and functional activation during the alcohol taste cue provides support for the hypothesis that lower white matter integrity in frontoparietal and corticolimbic networks is a factor in loss of control over alcohol consumption.

## Introduction

Alcohol is the most readily available and commonly abused drug across all age groups in the United States (Substance Abuse and Mental Health Services Administration 2010), making alcohol-related brain damage a pressing public health concern. In particular, white matter damage is a signature injury of alcohol use disorders (AUDs; Harper and Kril 1990; Kril and Halliday 1999). Evidence suggests that chronic alcohol abuse damages white matter on the cellular level by increasing oxidative stress (Crews and Nixon 2009; Fernandez-Lizarbe et al. 2009; Pascual et al. 2011) and downregulating genes critical to myelination (Lewohl et al. 2000; Liu et al. 2007). A recent meta-analysis of magnetic resonance imaging (MRI) studies comparing white matter volume in AUD and healthy control groups found a significant effect size of *g *=* *0.304 for the white matter volume deficit associated with AUD diagnosis (Monnig et al. 2012b). Individuals with AUDs exhibit neuropsychological impairment in complex functions that rely on intact white matter, including memory retrieval, visuospatial processing, and emotional regulation (Filley 2001; Moselhy et al. 2001; Oscar-Berman and Marinković 2007). Several studies have found associations between lower white matter integrity and poorer visuospatial processing, executive functioning, and memory in AUD (Pfefferbaum et al. 2006; Rosenbloom et al. 2009; Konrad et al. 2012; Trivedi et al. 2013). However, the relation between integrity of specific white matter tracts and alcohol cue processing in individuals with AUDs has not been investigated to date. This relationship may be especially important because heightened neural reactivity to alcohol cues is a reliable finding in AUDs (Schacht et al. 2013) and may be associated with increased craving or risk for alcohol relapse (Grüsser et al. 2004; Beck et al. 2012).

Diffusion tensor imaging (DTI) offers fine-grained analysis of white matter microstructure beyond volumetric measures and can detect abnormality prior to the onset of measureable atrophy (Fjell et al. 2008; Giorgio et al. 2010). DTI is based on the principle of Brownian motion of water molecules (Basser and Pierpaoli 1996) and allows inferences about the integrity of white matter to be made noninvasively. In general, optimal myelination of fibers results in increased directionality of water diffusion in white matter microstructure. The extent to which diffusion in a voxel is nonrandom, or anisotropic, is quantified in terms of fractional anisotropy (FA), a value ranging from 0, which corresponds to unrestricted diffusion, to 1, indicative of diffusion along a single axis. FA is believed to reflect multiple properties of white matter microstructure, such as axonal diameter, axonal density, myelination, and fiber bundle organization (Pierpaoli et al. 2001; Beaulieu 2002; Song et al. 2002, 2003, 2005), and is often reported as a summary index of white matter integrity.

The corpus callosum, the largest white matter tract, has shown reduced FA or abnormal diffusivity in several studies of AUDs (Pfefferbaum et al. 2000, 2002; Pfefferbaum and Sullivan 2005). A longitudinal study of alcohol-dependent individuals demonstrated reversal of white matter abnormality in the corpus callosum with a year of abstinence, suggesting that some abnormality found in AUDs is directly related to alcohol consumption (Alhassoon et al. 2012). In other brain regions, alcohol-dependent individuals with several months of abstinence showed lower FA than healthy control participants in the frontal forceps and superior cingulate, along with higher diffusivity in the fornix, internal and external capsule, and superior longitudinal fasciculus (Pfefferbaum et al. 2009). Another study of AUD individuals with 1 week of abstinence found lower FA in the external capsule, anterior and superior corona radiata, and thalamus relative to a control group (Yeh et al. 2009). Moreover, AUD individuals with at least 1 year of abstinence exhibited lower FA in the superior corona radiata, splenium of corpus callosum, internal capsule, posterior thalamic radiation, and sagittal striatum compared to a healthy group (Monnig et al. 2012a). Taken together, previous studies suggest that alcohol-related white matter abnormality occurs in broadly distributed white matter tracts, yet it may preferentially affect networks regulating motivation and reward salience (Harris et al. 2008; Pfefferbaum et al. 2009; Yeh et al. 2009).

Evidence of widespread white matter damage in AUDs raises questions about the functional import of these changes. A study that classified participants who completed AUD treatment as returning to heavy use or sustaining treatment gains at 6-month follow-up found significantly higher FA in frontal white matter at baseline in the treatment sustainers (Sorg et al. 2012). This association between baseline white matter integrity and treatment outcome suggests that the role of white matter in AUDs warrants further attention.

A possible mechanism relating white matter integrity to susceptibility to alcohol problems is that alcohol may disrupt top-down, behavioral regulation networks that modulate reactivity to environmental cues, including alcohol stimuli. This study approached this issue by examining the association between white matter integrity and neural reactivity to an alcohol taste cue in heavy drinkers. It has been hypothesized that alcohol affects the neuronal networks that underlie reward-based learning and executive control, both of which have been implicated in the development of substance dependence (Koob and Volkow 2010). At the network level, decreased white matter integrity may produce disconnection or otherwise alter function in cortical and subcortical reward substrates. In particular, alcohol may sensitize subcortical systems involved in reward or approach behavior while it dampens frontoparietal cortical networks important for self-regulation (Koob and Volkow 2010). Given findings of premorbid abnormality in white matter integrity and functional connectivity of frontoparietal and frontocerebellar networks, it seems likely that some structural and functional liabilities to problem drinking predate the use of alcohol (Herting et al. 2010, 2011; Wetherill et al. 2012). A model that takes into account both premorbid vulnerability to and direct effects of alcohol is consistent with models of addiction that describe an overactive incentive motivational network in conjunction with a compromised control network (Volkow et al. 2002; Kalivas and Volkow 2005; Baler and Volkow 2006; Wiers et al. 2007; Hutchison 2010).

Multimodal neuroimaging approaches that combine functional MRI (fMRI) and DTI are ideally suited to address the ramifications of white matter network abnormality. The objective of this study was to investigate the functional implications of individual differences in white matter integrity by testing whether FA values were related to blood-oxygenation-level-dependent (BOLD) response elicited by an alcohol taste cue in a sample of heavy drinkers. A previous investigation with a largely overlapping sample demonstrated that the alcohol taste cue was associated with increased BOLD response throughout networks involved in incentive motivation, with the magnitude of activation positively related to several indicators of alcohol problem severity (Claus et al. 2011). In line with the concept that disruption of white matter networks results in dysregulated alcohol cue processing, we predicted that lower white matter integrity in frontoparietal networks that participate in behavioral control would be associated with greater BOLD activation in response to the alcohol taste cue, particularly in subcortical reward processing substrates.

## Method

### Participants

#### Recruitment criteria

Inclusion and exclusion criteria have been described elsewhere (Claus et al. 2011). In brief, participants ranged in age from 21 to 56, had no contraindications for MRI scanning, and had no history of traumatic brain injury with loss of consciousness >5 min. The study recruited participants who reported at least five heavy-drinking episodes (≥4 drinks for women, ≥5 drinks for men on a single occasion) in the past month. Participants completed the Alcohol Use Disorders Identification Test (AUDIT; Babor et al. 2001), the Alcohol Dependence Scale (ADS; Skinner and Horn 1984), and the Impaired Control Scale (ICS; Heather et al. 1993). Drinks per drinking day, current smoking status, and use of other drugs were assessed with the 60-day or 90-day timeline follow-back (TLFB; Sobell and Sobell 1992). Participants who reported using marijuana were not excluded from the study. Participants with more than minimal use of drugs other than alcohol, tobacco, or marijuana in the past 60 days were excluded from the study. Of the final sample (*N *=* *332) included in analyses, data on drug use were available for 317 participants. Cigarette smoking was reported by 52% of participants. Thirty-seven percent of participants reported using marijuana, with frequency averaging 20% of days in the past 60 days. Fifteen participants (5%) reported occasional use of other illicit drugs in the past 60 days. Relations between alcohol use measures and white matter FA were assessed with Pearson correlations.

Participants were instructed to abstain from alcohol for 24 h prior to study procedures, and a blood alcohol content of zero was confirmed with a breathalyzer prior to scanning. All participants had a Clinical Institute Withdrawal Assessment Scale (Sullivan et al. 1989) score lower than 8, indicating no need for detoxification. Study procedures were approved by the Human Research Review Committee at the University of New Mexico, and study participants provided informed consent.

### Imaging protocols

#### Image acquisition

All MRI scans were collected on a 3T Siemens Trio (Erlangen, Germany) whole-body scanner. Prior to the acquisition of anatomical scans, localizer scans were acquired. An echo-planar, gradient-echo, pulse sequence (TR = 2000 msec, TE = 29 msec, flip angle = 75°) was acquired with a 12-channel head coil, and images were acquired parallel to the ventral surface of the participant's orbitofrontal cortex to reduce signal dropout and distortion in this region (Deichmann et al. 2003). Each volume consisted of 33 axial slices (64 × 64 matrix, 3.75 × 3.75 mm^2^, 3.5 mm thickness, 1 mm gap). In addition, a high-resolution T1-weighted 3D MP-RAGE anatomical image was acquired (TR = 2530 msec, TE = 1.64 msec, flip angle = 7°, 192 sagittal slices, 256 × 256 matrix, slice thickness = 1 mm) for each participant.

DTI scans were acquired via single-shot, spin-echo, echo-planar imaging (EPI) with a twice-refocused balanced echo sequence to reduce eddy current distortions. DTI data were collected along the AC/PC line, with FOV = 256 × 256 mm, 128 × 128 matrix, slice thickness of 2 mm (isotropic 2 mm resolution), NEX = 1, TE = 84 msec, and TR = 9000 msec. A multiple-channel radiofrequency (RF) coil was used, with GRAPPA (X2), 30 gradient directions, and *b* = 800 sec/mm^2^, and the *b* = 0 experiment was repeated five times (Jones et al. 1999).

**Figure 1:**
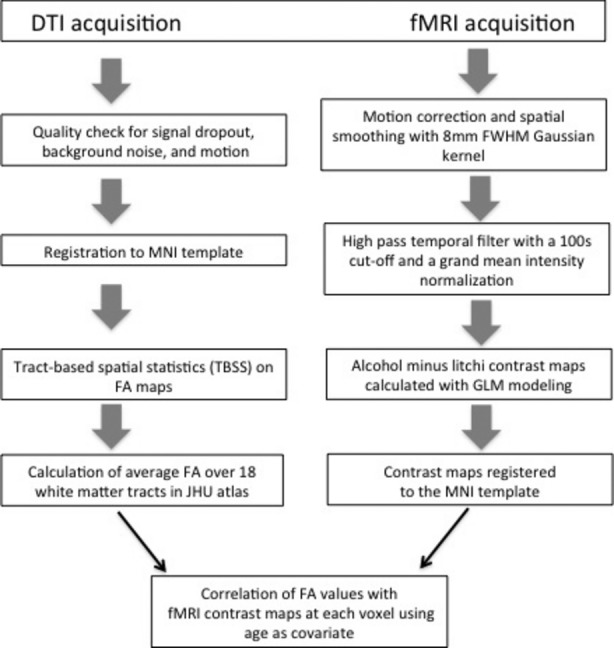
Schematic of data processing and analysis steps.

See Figure 1 for a schematic of data processing and analysis steps following image acquisition.

#### DTI analysis

DTI preprocessing entailed (1) data quality check, (2) motion eddy current correction, and (3) adjustment of diffusion gradient directions.

##### Data quality check

The DTI data were checked for: (1) signal dropout due to subject motion, producing striated artifacts on images; (2) excessive background noise in the phase encoding direction, due to external RF leakage in the MRI scan room or subject motion; and (3) large amounts of motion in the absence of signal dropout. A DTI volume was dropped if the motion was more than 4 mm of root mean square displacement. If more than 10% of gradient directions were dropped for any of the above reasons, then the subject was not considered for further analysis. Of 481 participants scanned, data for 145 were excluded by stringent quality control, leaving 336 participants with acceptable DTI data. Exclusions were typically due to participant motion or scanner noise. The data quality check considered the effects of motion in two ways. If the participant moved during acquisition of a specific brain volume, then the image quality was degraded. After the participant ceased movement and held still at a new location, the image quality would be good, but the motion registration algorithm would show a large displacement. The combined quality check criterion was highly stringent, as evidenced by its exclusion of 30% of participants who were scanned. The criterion of excluding the participant from further DTI analysis if 10% of the gradient directions were dropped was based on empirical findings from a previous study by our group (Ling et al. 2012).

##### Motion and eddy current correction

We registered all the images to a *b* = 0 sec/mm^2^ image. Twelve degrees of freedom, affine transformation with mutual information cost function was used for image registration.

##### Adjustment of diffusion gradient direction

Two corrections were applied to the diffusion gradients. The nominal diffusion gradient directions were prescribed in the magnet axis frame and rotated to correspond to the image slice orientation. No correction was required if the imaging slice was pure axial. A second correction accounted for any image rotation during the previous motion and eddy current correction step. The rotation part of the transformation found previously was extracted, and each gradient direction vector was corrected accordingly. Image registration and transformations steps were done with FMRIB's Linear Image Registration Tool (FLIRT), and the detection of outliers and data pruning was done with a custom program written in IDL (http://www.ittvis.com). Dtifit was used to calculate the diffusion tensor and the FA maps.

FA values were obtained using FMRIB Software Library (FSL), tract-based spatial statistics (TBSS; Smith et al. 2006). Each FA image was aligned to the standard-space FMRIB58 FA image (voxel size of 1 × 1 × 1 mm) with a nonlinear registration algorithm (FMRIB's Nonlinear Image Registration Tool, Oxford, UK). Following transformation to the target and affine transformation to MNI152 space, all FA images were merged into a single 4D image file, from which the FA skeleton was calculated using a threshold value of 0.2. White matter tracts were defined using the Johns Hopkins University, International Consortium for Brain Mapping DTI-81 labels atlas, with highest probability thresholding at 25% and white matter tractography atlas (Mori et al. 2005; Wakana et al. 2007). White matter tracts were selected for analysis on the basis of previous studies summarized above and hypotheses regarding the involvement of self-regulation and reward networks. These 18 tracts were as follows: genu, body, and splenium of corpus callosum; fornix; forceps minor; anterior, posterior, and retrolenticular limbs of internal capsule; anterior, superior, and posterior corona radiata; anterior and posterior thalamic radiations; sagittal striatum; external capsule; cingulate gyrus; superior longitudinal fasciculus; and inferior frontooccipital fasciculus. Within individuals, FA values for the skeletonized voxels that intersected with the tract atlas were averaged to obtain a single value per tract to be used in subsequent correlations with fMRI data. Right and left hemispheres were averaged for the 13 bilateral tracts, as hypotheses did not stipulate differential effects based on hemisphere.

#### fMRI task and analysis

To measure cue-elicited responses to alcohol, we used a task described previously in which a small amount of the participant's preferred alcoholic beverage was alternated with litchi juice, an appetitive control (Filbey et al. 2008; Claus et al. 2011). All analyses were completed using tools from FSL and are described in detail in Claus et al. (2011). An overview is shown in Figure 1. The contrast of interest compared the alcohol cue minus the juice cue. Contrast maps from individual subject analyses were registered to the MNI152 template in a two-step registration process using registration parameters from the registration of the mean EPI image to the individual subject's T1 image, and the registration of the T1 to the MNI152 template. All steps used FLIRT.

#### FA-BOLD correlations

Correlations between averaged FA values and the whole-brain contrast maps (alcohol minus litchi) from the alcohol cue task were analyzed to identify task-related regions that were significantly associated with white matter integrity. This step was done for each FA map by a linear regression of the averaged FA values with each voxel of the BOLD contrast maps across the subjects. Age is associated with FA decreases independent of alcohol intake (Giorgio et al. 2010; Michielse et al. 2010) and could be a potential confound. In this sample, a linear, negative correlation was observed for age and averaged FA for 15 of 18 of the tracts of interest, with significant Pearson's *r*'s ranging from −0.128 to −0.472. Because of the relation between FA and age, which was consistent with the aging literature cited above, age was included as a covariate in regression analyses. However, results did not change appreciably when age was not included as a covariate. Four participants with acceptable DTI data did not have fMRI taste task data and were excluded from analysis. In addition, 15 participants had FA values that were outliers of >3 standard deviations (SDs) on at least one white matter region of interest (ROI). Outliers on a given ROI were excluded for that analysis, and the number of excluded participants ranged from 0 to 7 for the 18 white matter ROIs. For each correlation, the thresholded image was corrected for multiple comparisons using cluster-based thresholding as implemented in FSL, with a voxel-wise threshold of *z *>* *2.3 and a cluster threshold of *P *<* *0.05.

## Results

The sample included 332 individuals (102 female, 230 male) with a mean of 31.2 (SD = 9.4) years of age. Table 1 summarizes demographic and clinical characteristics of the sample. On average, participants endorsed a moderate level of alcohol problem severity on the ADS and AUDIT. On the ADS, 64% of the sample scored ≥9, reflecting a high likelihood of diagnosis of alcohol dependence (Allen and Wilson 2003). Moreover, 96% of participants scored ≥8 on the AUDIT, indicating hazardous drinking and possible dependence (Babor et al. 2001).

**Table 1:** Demographic and clinical characteristics (*N *=* *332).

	*n* providing data	*M* (SD) or percent
Age	332	31.2 (9.4)
Education (years)	290	14.4 (2.5)
Alcohol Dependence Scale (ADS)	308	13.1 (8.1)
Alcohol Use Disorder Identification Test (AUDIT)	308	18.5 (7.7)
Impaired Control Scale (ICS)	310	44.5 (21.3)
Number of years drinking regularly (years drink)	310	12.0 (8.9)
Average drinks per drinking day (DPDD)	302	7.0 (4.2)

**Figure 2:**
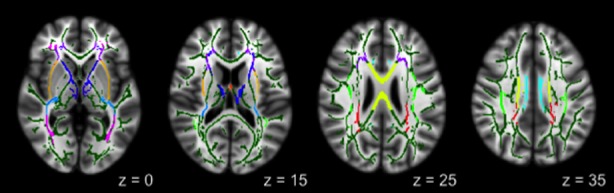
Atlas-based regions of interest showing significant correlations with BOLD response: anterior corona radiata (ACR; purple); anterior thalamic radiation (ATR; blue); external capsule (EC; light orange); retrolenticular part of the internal capsule (RLIC; light blue); inferior frontooccipital fasciculus (IFOF; magenta); fornix (FNX; orange); body of the corpus callosum (yellow); posterior corona radiata (PCR; red); cingulate gyrus (CG; cyan); and superior longitudinal fasciculus (SLF; light green).

White matter tracts showing significant correlations between averaged FA and BOLD were the anterior corona radiata, anterior thalamic radiation, body of corpus callosum, cingulate gyrus, external capsule, fornix, inferior frontooccipital fasciculus, posterior corona radiata, retrolenticular limb of internal capsule, and superior longitudinal fasciculus (Fig. 2). With the exception of the anterior thalamic radiation, all correlations were negative, indicating that lower FA was associated with greater BOLD response.

Pearson correlations between averaged FA values for the significant tracts and alcohol use measures are shown in Table 2. White matter integrity was negatively related to measures of alcohol use severity and duration, with correlations of modest magnitude. Anterior corona radiata, cingulate gyrus, fornix, and inferior frontooccipital fasciculus consistently showed significant relations with alcohol use measures. Of the measures, number of years of drinking and drinks per drinking day were related most frequently to FA.

**Table 2:** Bivariate correlations of white matter ROIs with alcohol use measures.

	AUDIT	ADS	ICS	Years drink	DPDD
ACR	−0.213**	−0.168**	−0.213**	−0.374**	−0.263**
ATR	−0.078	0.034	0.015	−0.067	−0.075
BCC	−0.109	−0.087	−0.159**	−0.281**	−0.079
CG	−0.201**	−0.113	−0.158**	−0.156**	−0.135*
EC	−0.073	0.025	0.020	0.012	−0.085
FNX	−0.268**	−0.301**	−0.305**	−0.382**	−0.209**
IFOF	−0.216**	−0.167**	−0.192**	−0.388**	−0.224**
PCR	−0.111	−0.090	−0.109	−0.220**	−0.184**
RLIC	−0.140*	−0.057	−0.053	−0.144*	−0.130*
SLF	−0.145*	−0.109	−0.114	−0.248**	−0.160**

*n*'s range from 289 to 296.

* *P *<* *0.05;

** *P *<* *0.01.

**Figure 3:**
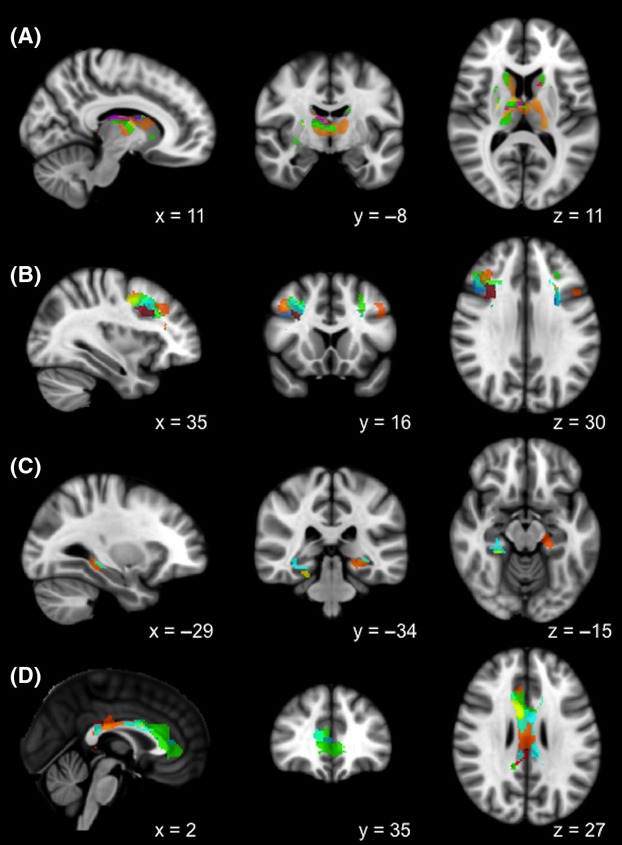
Overlapping clusters of BOLD activation in the (A) thalamus and caudate, (B) medial frontal gyrus, (C) parahippocampal gyrus, and (D) cingulate gyrus, correlated with FA in the anterior corona radiata (ACR; purple); anterior thalamic radiation (ATR; blue); external capsule (EC; light orange); retrolenticular part of the internal capsule (RLIC; light blue); inferior frontooccipital fasciculus (IFOF; magenta); fornix (FNX; orange); body of the corpus callosum (yellow); posterior corona radiata (PCR; red); cingulate gyrus (CG; cyan); and superior longitudinal fasciculus (SLF; light green); masks from anatomical atlases were applied to highlight the areas of specific overlap.

**Figure 4:**
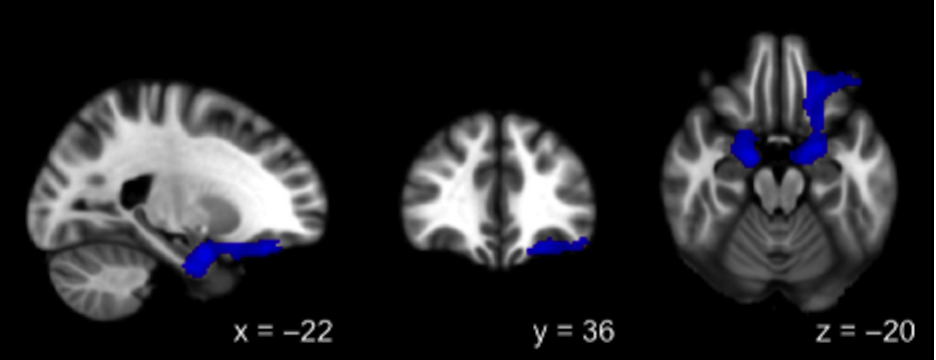
Positive correlation between BOLD activation and FA in the anterior thalamic radiation (ATR).

Regions on the BOLD contrast maps where greater cue reactivity was associated with lower averaged FA included the medial frontal gyrus, cingulate gyrus, precuneus, parahippocampal gyrus, fusiform gyrus, insula, thalamus, putamen, caudate, and cerebellum (Fig. 3). The positive correlation noted above was between FA of the anterior thalamic radiation and BOLD response in the orbitofrontal cortex, amygdala, pons, and parahippocampal gyrus (Fig. 4). Clusters with significant correlations are listed in Table 3.

**Table 3:** White matter tracts with locations of significantly correlated clusters of BOLD activation.

			*Z*-max coordinates (mm)				
WM tract	Cluster size (voxels)	*Z*-max	*X*	*Y*	*Z*	Brodmann areas	Anatomical region(s)
ACR	1854	3.61	−20	4	32	3, 6, 24, 31, 32	L medial frontal gyrus L postcentral gyrus L cingulate gyrus
ATR*	1994	4.24	−20	−2	−24	11, 34, 47	L orbitofrontal cortex L inferior frontal gyrus R, L parahippocampal gyrus R, L amygdala Pons
BCC	3911	3.54	30	0	38	6, 7, 24, 31	R medial frontal gyrus R cingulate gyrus R thalamus R, L precuneus
	2389	4.16	34	−54	2	19, 37	R parahippocampal gyrus R fusiform gyrus R medial temporal gyrus
CG	13,511	4.54	34	−54	4	18, 19, 24, 30, 37	R cingulate gyrus R fusiform gyrus R, L parahippocampal gyrus R lingual gyrus R, L thalamus R caudate R, L cerebellum
EC	5262	3.82	−2	−82	−8	18, 30	R, L posterior cingulate R, L cuneus R, L lingual gyrus R, L cerebellum
	2083	3.28	−22	−24	56	3, 7, 24, 31	L cingulate gyrus L precentral gyrus L precuneus
FNX	11,306	4.65	14	−6	12	8, 9, 10, 13, 24, 27, 32	R, L medial frontal gyrus R, L superior frontal gyrus R insula R anterior cingulate R cingulate gyrus R, L thalamus R, L caudate L parahippocampal gyrus
	1881	3.44	32	−78	−36		R, L cerebellum
IFOF	1956	3.70	−20	0	32		L postcentral gyrus L caudate L putamen
	1700	3.16	26	−8	20		R thalamus
PCR	4257	3.66	−20	4	32	23, 24	L putamen R, L cingulate gyrus R posterior cingulate
RLIC	2020	3.85	34	−52	2		R thalamus
	1656	3.64	−32	0	22	24	L cingulate gyrus
SLF	16,354	4.40	−20	4	34	8, 9, 13, 24, 31, 32	R, L superior frontal gyrus R, L medial frontal gyrus R, L cingulate gyrus R, L anterior cingulate R caudate R, L putamen R insula R, L thalamus

ACR, anterior corona radiata; ATR, anterior thalamic radiation; BCC, body of corpus callosum; EC, external capsule; CG, cingulate gyrus; FNX, fornix; IFOF, inferior frontooccipital fasciculus; PCR, posterior corona radiata; RLIC, retrolenticular limb of internal capsule; SLF, superior longitudinal fasciculus.

* The correlation between ATR and BOLD activation was positive, whereas all other correlations were negative.

## Discussion

This study investigated the functional implications of white matter integrity in the context of heavy alcohol consumption by correlating FA values of 18 white matter tracts with BOLD activation during an alcohol cue. FA values of 10 tracts subserving frontoparietal and corticolimbic networks showed significant correlations with BOLD response to the taste of alcohol. All but one of these correlations were negative, supporting the prediction that lower white matter integrity would be related to heightened response to the alcohol cue.

Evidence from a variety of imaging paradigms has implicated abnormalities of connections among the thalamus, basal ganglia, limbic system, and cerebral cortex in substance abuse and dependence. The results of this study offer converging evidence that white matter connections among cortical and subcortical substrates of sensorimotor processing, reward learning, and higher level cognition participate in the development and maintenance of problematic alcohol use. A candidate mechanism in the development of substance dependence is the failure of top-down systems of self-regulation and effortful processing, particularly frontoparietal networks, to override subcortical networks involved in habitual responses to reward cues, which are strengthened with accumulated exposure to drug cues and consumption (Koob 2006; Koob and Volkow 2010). Given the age range of our sample (ages 21–56), it is important to recognize that changes in FA and downstream changes in the function of neural networks may begin relatively early in the trajectory of problem drinking.

Lower FA consistently showed significant correlations with greater BOLD activity in the thalamus, medial frontal gyrus, cingulate, and parahippocampal gyrus. One interpretation of this pattern is that individuals exhibit greater cue reactivity when bottom-up activity originating in the thalamus guides subsequent attentional orienting and salience attribution in the prefrontal cortex and limbic system. A great body of literature has demonstrated the role of prefrontal cortex and cingulate in affective and reward-related decision making (Bechara et al. 1998; Bechara 2004; Rogers et al. 2004; Cohen et al. 2005). Because participants were not engaged in a decision-making task, it remains unknown how increased cue reactivity might affect these processes. However, one hypothesis for future study is that lower integrity of frontoparietal white matter networks mediates the relationship between increased cue reactivity and alcohol urges. It should be noted that white matter fiber tracts are largely bidirectional and that analyses did not investigate the temporal sequence of activation. Thus, an alternative mechanism that might operate instead of or in conjunction with weakened top-down control over bottom-up response is alteration of the signal communicated upward from subcortical to cortical regions as a result of changes in white matter integrity, affecting the processing of cue-eliciting stimuli.

A recent meta-analysis of alcohol cue reactivity found that heavy drinkers reliably showed increased activation in the right caudate, cingulate cortex, thalamus, and ventromedial prefrontal cortex relative to control cue conditions (Schacht et al. 2013). However, alcohol-elicited activation in these areas was not significantly greater in heavy-drinking groups compared to control groups, suggesting that the incentive salience of alcohol cues may be comparable across groups (Schacht et al. 2013). Areas that did differentiate AUD and control groups were the bilateral precuneus, left posterior cingulate, and left superior temporal gyrus. The findings of the current study converge with the meta-analytic findings, which demonstrated the importance of cue-elicited activity in posterior regions such as the precuneus and posterior cingulate in differentiating alcohol dependent from healthy individuals and varying as a function of severity. The current results provide additional support for the involvement of posterior regions in cue reactivity, as white matter tracts traversing the body of the corpus callosum, cingulum bundle, and external capsule all have significant projections to the posterior cingulate, precuneus, and/or other regions within the posterior parietal lobe (Hofer and Frahm 2006; Fernández-Miranda et al. 2008a,b2008b; Fortin et al. 2012; Jones et al. 2013). Importantly, these tracts also project to regions known to respond during cue-elicited craving, including the supplementary motor area, medial frontal cortex, insula, and dorsal striatum (Claus et al. 2011; Schacht et al. 2013).

In addition to posterior cortical regions, we also found significant, inverse correlations between white matter integrity and BOLD response in frontal regions including the inferior, medial, and superior frontal gyri. Lateral frontal regions typically have been implicated in cognitive control and goal-directed behavior. Given that response to alcohol cues in the dorsolateral prefrontal cortex and medial frontal gyrus has been positively associated with alcohol problem severity (Claus et al. 2011), our findings could be interpreted as providing further evidence of engagement of these regions in individuals with more extensive drinking histories. The negative correlation of BOLD activity in these regions with white matter integrity suggests the possibility that, although these regions may come online to a greater degree during alcohol cue presentation, lower white matter integrity in tracts that project to limbic and temporal regions (e.g., fornix, cingulate, and superior longitudinal fasciculus) may result in less effective control over representations in bottom-up processing streams. Notably, the fornix and cingulate are consistently implicated in studies of alcohol dependence (Schulte et al. 2010). A caveat to these interpretations is that several tracts, such as the superior longitudinal fasciculus, are quite large and are known to incorporate several subcomponents (Fernández-Miranda et al. 2008a; Schmahmann et al. 2008). Future studies examining the relation of cue reactivity to specific subtracts would be useful.

A recent study found that alcohol-dependent participants had lower gray matter volume of lateral frontal, medial frontal, and parietal-occipital clusters compared to healthy control participants and that volume of the medial frontal and parietal-occipital clusters significantly predicted time to relapse, after controlling for age, IQ, years of alcohol use, and consumption over the 90 days preceding treatment (Rando et al. 2011). The clusters that predicted relapse in that study were consistent with the clusters of BOLD activity in the anterior and posterior cingulate, precuneus/cuneus, and medial prefrontal cortex associated with lower FA in our study. These findings underscore the importance of posterior parietal and medial frontal gray matter regions and their white matter connections in regulating neural response to alcohol cues.

The single positive correlation was found between the anterior thalamic radiation, which connects the anterior and dorsomedial thalamic nuclei with the prefrontal cortex, and BOLD activity throughout the amygdala, prefrontal cortex, and parahippocampal gyrus. Involvement of the amygdala is particularly interesting because it receives direct input from the olfactory bulb, which presumably would be a primary sensory substrate for the alcohol taste cue. Frank and Claus (2006) put forward a model of striato-orbitofrontal interaction in which the orbitofrontal cortex receives input from the amygdala about reinforcement value of outcomes associated with sensory cues. The orbitofrontal cortex, which in turn projects to the basal ganglia, encodes and maintains in working memory information about reward to enable adaptive, differential responding (Frank and Claus 2006). The positive correlation invites speculation that repeated experiences with alcohol selectively strengthens the influence of subcortical outputs to prefrontal cortex and limbic structures through enhanced white matter connectivity, possibly increasing the relative influence of subcortical pathways over subsequent reward-seeking behavior. Again, given that the anterior thalamic radiation is a bidirectional tract, the direction and order of effects remain unknown.

Although white matter damage has been established as a hallmark injury of AUD, causal mechanisms are still under investigation. In animal models of alcohol dependence, the presence of alcohol in the brain triggers stimulation of proinflammatory cascades leading to cell death or dysfunction and inhibition of neurogenesis in adult neural stem cells in the olfactory bulb and hippocampus (Crews and Nixon 2009). Pinpointing mechanisms of alcohol-induced brain damage in vivo in humans presents a challenge, but our findings support the notion that long-term heavy drinking contributes to decreased white matter integrity. Alcohol-related white matter damage is likely to be one constituent of the AUD cycle in which heavy drinking contributes to impaired cognition and emotion regulation, leading to further problematic drinking (Crews 1999). A potential clinical implication is that those with long-standing AUD, and therefore greater damage to white matter substrates, may have more difficulty applying cognitive or emotional-regulation strategies in the context of AUD intervention.

A strength of this study is its use of multimodal imaging methods to explore functional correlates of white matter integrity in problem drinking. Further strengths are the size of the sample and the variability in participants’ drinking histories. A major limitation is the inherent inability of cross-sectional design to establish causal relations between white matter profiles and cue reactivity. Whether greater cue reactivity preceded or followed heavy drinking remains a topic for further investigation. In the absence of a control group, we cannot infer that relations among white matter tracts and BOLD activation are unique to the heavy-drinking population. Moreover, although DTI has been instrumental in increasing understanding of the brain's structural connectivity, its limitations as an indicator of white matter integrity must be acknowledged. DTI metrics are influenced directly and indirectly by multiple properties of white matter and surrounding tissue, and research into precise mechanisms of change in these metrics in humans is ongoing. In addition, methods such as higher order fitting to address possible effects of crossing fibers were not used in this study. Because correction for multiple comparisons was not highly conservative, interpretation of findings should be cautious pending replication of these results in an independent sample.

Importantly, the cross-sectional design does not speak to whether individual variability in white matter profiles was a function of drinking history or a premorbid characteristic. A previous study found lower FA in several regions of interest shared with this study in alcohol-naïve adolescents with family history of alcohol dependence compared to healthy control participants (Herting et al. 2010). Moreover, lower FA was significantly related to reduced frontocerebellar functional connectivity (Herting et al. 2011). Taken together with a report of reduced functional connectivity in frontoparietal networks in a study of a similar adolescent sample (Wetherill et al. 2012), it appears that genetic liability for AUDs may account for a substantial proportion of variance in neural processing of alcohol cues.

Regarding sampling methodology, the decision to include participants based on quantity and frequency of recent drinking rather than diagnosis of alcohol abuse or dependence can be seen as an asset or a shortcoming, depending on perspective. The objective was to link neurobiological outcomes to overt behavioral rather than syndromal markers in order to increase generalizability to the population of heavy drinkers, who may or may not endorse diagnostic criteria. Related to this issue is the fact that a large minority of participants reported using drugs other than alcohol. This characteristic limits interpretation of findings, as the possible neurobiological effects of these other drugs were not evaluated. At the same time, the rate of illicit drug use in our sample was similar to the rate of 31% reported by heavy drinkers in a recent epidemiological sample (Substance Abuse and Mental Health Services Administration 2012), lending ecological validity to our findings.

In conclusion, our results expand on previous studies by establishing a relationship between lower white matter integrity and increased functional activation to an alcohol taste cue in a sample of heavy drinkers. Lower white matter integrity in the context of heavy drinking may entail dysregulation of neural response to alcohol cues in frontoparietal and corticothalamic networks governing reward salience and self-regulation.
